# Comparison of Vibration-Assisted Scratch Characteristics of SiC Polytypes (3C-, 4H- and 6H-SiC)

**DOI:** 10.3390/mi13040640

**Published:** 2022-04-18

**Authors:** Wuqing Lin, Zhongwei Hu, Yue Chen, Yuqiang Zhang, Yiqing Yu, Xipeng Xu, Jie Zhang

**Affiliations:** 1 Institute of Manufacturing Engineering, Huaqiao University, Xiamen 361021, China; vince_lin@sanan-ic.com (W.L.); chenyue@stu.hqu.edu.cn (Y.C.); 21011080019@stu.hqu.edu.cn (Y.Z.); xpxu@hqu.edu.cn (X.X.); 2 Institute of Mechanical Engineering and Automation, Huaqiao University, Xiamen 361021, China; yyqing@hqu.edu.cn; 3 Hunan Sanan Semiconductor Co., Ltd., Changsha 410000, China; jack_zhang@sanan-ic.com

**Keywords:** molecular dynamics method, SiC polytypes, vibration-assisted scratch, scratch characteristics

## Abstract

Single-crystal silicon carbide (SiC) is widely used because of its excellent properties. However, SiC is a typical hard and brittle material, and there are many challenges in realizing its high efficiency and high-precision machining. Grinding is the main method used to achieve the high-efficiency processing of SiC, but the contradiction between processing quality and processing efficiency is prominent. Vibration-assisted grinding is an effective method to realize high-efficiency and precision machining of SiC. To reveal the vibration-assisted grinding mechanism of SiC, the vibration-assisted nano-scratch process is studied using the molecular dynamics method, and the material removal process and damage formation mechanism in the vibration-assisted scratch are analyzed. Aiming at the three main structural crystal types, 3C-, 4H- and 6H-SiC, scratch simulations were carried out. The vibration-assisted scratch characteristics of SiC polytypes were evaluated from the perspectives of scratch force and the amorphous layer. It was found that the effects of vibration-assisted scratch on different crystal structures of SiC differ, and 3C-SiC is quite different from 4H- and 6H-SiC. Through vibration-assisted scratch simulations under different scratch conditions and vibration characteristics, the influence laws for machining parameters and vibration characteristic parameters were explored. It was found that increasing the frequency and amplitude was beneficial for improving the machining effect. This provides a basis for vibration-assisted grinding technology to be used in the high-efficiency precision machining of SiC.

## 1. Introduction

Silicon carbide (SiC) has excellent physical and chemical properties, such as good thermal conductivity and high temperature resistance, and is not easy to corrode, meaning that its semiconductor devices are widely used in new energy vehicles, aerospace and other emerging fields [1]. SiC also has the characteristics of high hardness, strength and brittleness and low fracture toughness. It is a typical difficult-to-machine material [2], which limits its wide application. In the actual machining process, due to the high hardness and brittleness of SiC, it is difficult to meet the requirements of high efficiency and low damage [3].

Vibration-assisted grinding is a process in which a certain amplitude is applied to the tool, combined with conventional grinding, to machine the workpiece [4,5]. Through an analysis of the vibration-assisted machining of hard and brittle materials, it was found that, compared with conventional grinding, the force shows a decreasing trend [6,7]; with the increase in vibration amplitude, the grinding force shows a nonlinear reduction [8]. Under the same processing parameters, the average cutting area of abrasive grains decreased and the grinding temperature could be reduced by about 8%; at the same time, vibration-assisted grinding could avoid the occurrence of tensile stress [9]. Vibration machining can significantly improve the surface quality [10] and the subsurface quality [11]. Studies have also shown that vibration-assisted machining is beneficial for improving tool blockage, reducing abrasive wear and improving the wear resistance of a grinding wheel [11,12]. Therefore, the vibration-assisted machining of SiC is an excellent method for avoiding the problems that may occur in conventional machining. In order to explore the complex grinding process in more depth, studying a single abrasive grain scratch can effectively explain various physical phenomena in the grinding process [13]. Chen [14] found that the scratch force periodically changed with the change in abrasive grain trajectory using molecular dynamics simulations. Cao [15] considered that the impact of the vibrating tool and cutting effect on the machined surface are the main reasons for material removal, and vibration application is conducive to improving the machining efficiency. Vibration-assisted scratch (VS) reduces the size of brittle fracture, and the periodically changing contact area and force between abrasive grains and the workpiece can promote the propagation of cracks in different directions. Qiao [16] found that VS can increase the critical load of brittle plastic transformation, promote the propagation of lateral cracks to the surface and is conducive to material removal. In addition, the critical depth of brittle plasticity of VS is greater than that of conventional scratch (CS) [17].

Due to SiC’s excellent properties, its material properties and processing process have attracted much attention. SiC is formed by the orderly arrangement and combination of Si atoms and C atoms; there are more than 200 crystal structures of SiC, including 3C-, 4H- and 6H-SiC. 3C-SiC is cubic crystal, also known as β-SiC, and its arrangement is usually described by ABCABC. 4H- and 6H-SiC are called α-SiC and have a hexagonal structure, in which the atomic arrangement of 4H-SiC is ABCB (or ABAC), and the atomic arrangement of 6H-SiC is ABCACB, as shown in Figure S1. [18]. The atomic arrangement of different crystalline SiCs directly leads to their different material properties. Yang [19] studied the mechanical properties of SiC polytypes through the first principles; the results show that the comprehensive mechanical properties of 6H-SiC are better than those of other crystalline SiC. Saurav Goel [20] concluded, through an empirical formula calculation, that 3C-SiC has the largest fracture toughness and hardness, 6H-SiC has the lowest hardness and fracture toughness is consistent with 4H-SiC. Xu [21] used first principles to study the mechanical and thermodynamic properties of SiC polytypes. The results show that 3C-SiC has obvious linear expansion anisotropy and elastic anisotropy, and the elastic anisotropy of 6H-SiC is the smallest. Kamitani [22] found that the room-temperature elastic constants of 4H- and 6H-SiC were similar. L.Pizzagalli [23] calculated the stability and mobility characteristics of screw dislocations in 4H-, 2H- and 3C-SiC and found that there were qualitative differences between the cubic system and hexagonal system in the slip plane. Kazuaki [24] calculated the total energy of SiC. The order was about 4H- < 6H- < 3C-SiC, and the total energy was related to the structural stability. Defects also show different characteristics on the SiC of different crystal structures [25], which also have different effects on subsequent machining.

The different material properties of different crystalline SiC, especially mechanical properties, lead to different machining effects. Some scholars have studied the efficiency and accuracy of SiC polytypes after machining, using experiments and simulations. Luo [26] used molecular dynamics simulation to simulate the conventional scratch process, and he believed that 3C-SiC was the most difficult to process, followed by 4H-SiC, and 6H-SiC was the least difficult. Tian [27] simulated the indentation and scratch on the different crystal faces of 4H- and 6H-SiC. There was no evident difference between 4H- and 6H-SiC in the indentation simulation, and 4H-SiC had a greater normal resistance than 6H-SiC in the scratch simulation; meanwhile, C-face may be easier to process than Si-face. Lu [28] carried out mechanical planarization polishing of the different crystal faces of 4H- and 6H-SiC, respectively, and found that the surface roughness of 4H-SiC was slightly larger than that of 6H-SiC, and the material removal rate was slower than that of 6H-SiC; the material removal rate of C-face was better than that of Si-face. However, Chen [29] believes that the material removal rate of Si-face was larger than that of C-face in chemical mechanical polishing. This shows that the material removal mechanisms of SiC polytypes and different crystal planes are very complex, and different machining methods will have different effects on the machining effect. According to the current research results, it can be inferred that the effect of vibration-assisted scratch will differ on different SiC polytypes and different crystal faces of SiC.

Therefore, it is necessary to conduct a systematic and comprehensive study on the vibration-assisted scratch (VS) of SiC polytypes (3C-, 4H- and 6H-SiC) and conduct convention scratch (CS) and VS simulations on the Si- and C- face of 3C-, 4H- and 6H-SiC, respectively. The scratch characteristics of SiC polytypes were compared using both CS and VS, and the responses of different crystal structures to vibration were studied to reveal the influence mechanism of VS on SiC polytypes.

## 2. Molecular Dynamics Simulations Model

Due to the limited materials and equipment, it was difficult to carry out the experiment. For the scratch simulation of single abrasive grain, the common simulation methods are molecular dynamics simulation (MD), finite element method (FEM) and smooth particle hydrodynamics method (SPH). The three methods focus on different aspects because of their own advantages and disadvantages. The simulation scales of FEM and SPH are larger than MD, which can study micro phenomena, such as cracks propagation. The MD is based on the atomic arrangement for modeling, which will have a deeper understanding of the material properties and reflect the anisotropy of materials. In this paper, the scratch simulation of SiC with different crystal structures is carried out. MD can fully consider the atomic arrangement structure to reflect the different material characteristics of different crystalline SiC. Scholars have conducted single abrasive grain scratch simulation using the MD method to study the surface formation mechanism, subsurface damage and material removal mechanism of SiC polytypes, and have made good progress [30,31,32,33].

**Figure 1:**
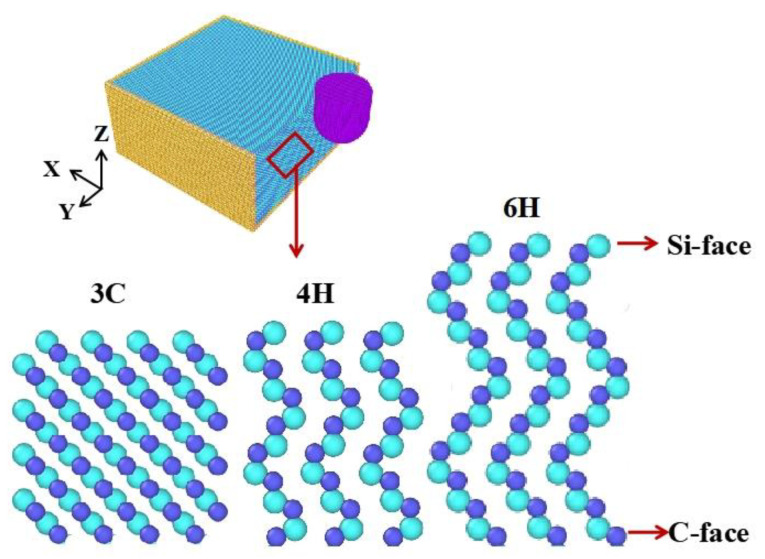
Molecular dynamics model of 3C-, 4H- and 6H-SiC.

To reveal the machining mechanism of VS process of SiC, the responses of SiC polytypes to vibrations in the VS process were studied. MD simulation was used to simulate the VS of single-crystal SiC. Firstly, the models of SiC polytypes were established according to the different stacking modes of atoms, as shown in Figure 1. The workpiece size was 30 nm × 30 nm × 15 nm. The workpiece model included boundary, thermostat and Newtonian atoms layers. During MD simulation, the atoms of thermostat atoms layer were kept at 293 K. The abrasive grain material was a diamond atom with perfect lattice. The shape was a combination of hemisphere and cylinder. The radius of the hemisphere was 4 nm and the height of the cylinder was 6 nm. All MD simulation experiments were completed by large-scale atomic/molecular massively parallel simulators (LAMMPS) [34], and the visualization of simulation data and the generation of snapshots were realized by OVITO software [35]. It is important to select the appropriate potential function to ensure the accuracy of results. Tersoff potential function [36] can accurately describe the interaction between atoms of covalent systems such as C and Si. Therefore, the Tersoff potential function of the polyatomic system was used to simulate the scratching process of SiC. In this model, the size effect is not considered [37]; when workpiece sizes are tens of nanometers, material properties are considered to be close to infinite surfaces. By comparing the previous research of conventional scratch SiC polytypes [27,28,30,32,38,39,40], it can be found that the change trend and magnitude of force are consistent, and the morphology and thickness of the amorphous layer are also very close, which can prove that the MD models in this paper are suitable to simulate the CS and VS of SiC polytypes.

In the scratching process, the abrasive grain scratched the surface of the workpiece on the Si-face ((0001) plane) and C-face ((000-1) plane) of 3C-SiC, 4H-SiC and 6H-SiC, respectively. Considering the technical, time and cost factors, the timestep was set as 1 fs. Based on the established simulation model, the depth of cut was set to 3, 3.5, 4, 4.5 nm, the speed was set to 60, 80, 100, 120 m/s, the VS simulation model was established and the amplitude was set to 1, 2, 3, 4, 5 nm, with frequencies of 4.17, 8.33, 16.67, 33.33 and 66.67 GHz. When changing other parameters, the depth of cut was maintained at 4 nm, the speed at 100 m/s, the amplitude at 0 nm (CS) or 4 nm and the frequency at 16.67 GHz.

## 3. Results and Discussion

After vibration was applied, the response of SiC polytypes and different crystal plane of SiC to vibration application differed. Different amplitude, frequency, scratch depth and speed have different effects on the vibration-machining effect of different crystal structures of SiC, which can significantly reduce the tangential force (F_x_) and normal force (F_z_), increase lateral force (F_y_), increase the atomic number of the amorphous layer, that is, the volume of the amorphous layer (V), and reduce the thickness of the amorphous layer (D). In this paper, the effect of vibration will be evaluated from the aspects of scratch force and the amorphous layer.

### 3.1. Scratch Force Analysis

**Figure 2:**
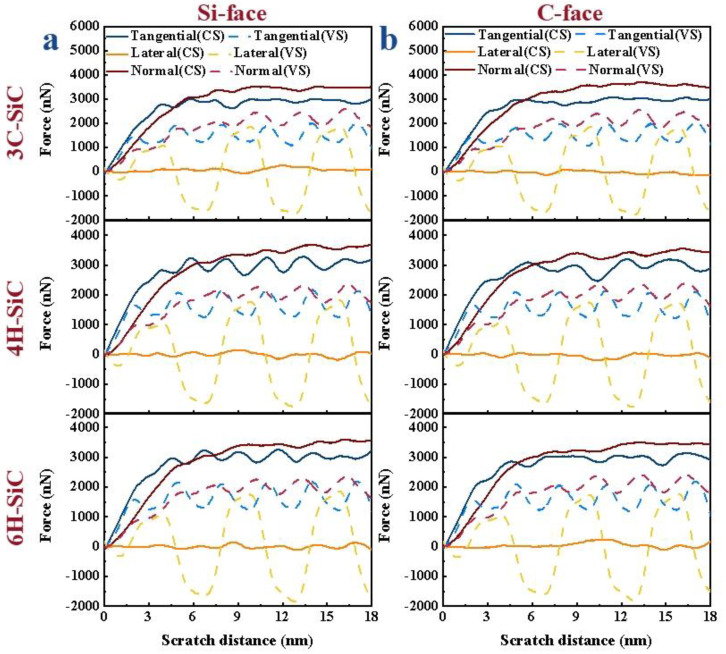
Comparison of conventional and vibration-assisted scratch forces of SiC polytypes: (**a**) the Si-face of 3C-, 4H-and 6H-SiC, respectively, (**b**) the C-face of 3C-, 4H-and 6H-SiC, respectively, the simulation parameters of conventional scratch are 4 nm (depth) and 100 m/s (scratch speed) and the simulation parameters of vibration-assisted scratch are 4 nm (depth), 100 m/s (scratch speed), 4 nm (amplitude) and 16.67 GHz (frequency).

Scratch force is an important parameter, and its size has a great impact on surface quality and tool life. To reflect the change trend of scratch force, the force–displacement curves were processed; the processing method can be found in Reference [7]. It can be seen from Figure 2 that the F_x_ and F_z_ gradually increase with the increase in scratch distance. When the scratch distance reached 6 nm, the machining process gradually tended to be stable after the abrasive grains completely entered the workpiece, so that the F_x_ and F_z_ gradually tended to be stable. In the subsequent stable scratching process, the F_x_ and F_z_ of CS remained basically unchanged, while the F_x_ and F_z_ of VS fluctuated periodically with the vibration of movement trajectory, but were smaller than the F_x_ and F_z_ of CS. In the CS, the F_y_ was almost zero and, with the application of vibration, the F_y_ showed positive and negative periodic changes. The change period was consistent with the vibration period of abrasive grains. It can also be seen from Figure 2a,b that the crystal structures and crystal plane of SiC will not affect the overall change trend of scratch force in the scratch process.

To further compare the scratch force in the CS and VS under different parameters, the scratch force within 6–15 nm in the steady state was selected for average calculation to obtain the average F_x_, F_z_ and F_y_ during CS and VS. Then, the reduction ratios of the scratch force in the VS to that in the CS at the same scratch depth and speed were calculated. The reduction ratio of the scratch force can be used to evaluate the effect of VS.

**Figure 3:**
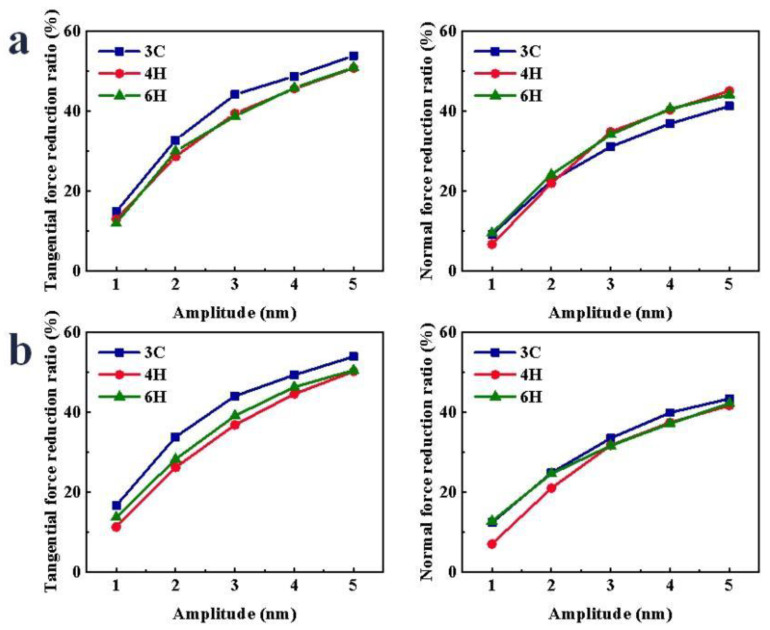
Variation in scratch force reduction ratio of SiC polytypes with different amplitudes: (**a**) Si-face and (**b**) C-face of SiC.

During the VS process, the variations of scratch force under different amplitudes are shown in Figure S2. With the increase in the amplitude, the displacement in the Y direction increased. Therefore, the acceleration of the abrasive grain in the Y-direction was greater, and the impact effect was greater, so the F_x_ and the F_z_ gradually decreased. During the CS process, the F_y_ was almost zero. After the vibration was applied, the movement of the abrasive grain in the Y-direction led the F_y_ to significantly increase. As shown in Figure 3, after the vibration was applied, the F_x_ and F_z_ in the scratch process were significantly reduced. The reduction ratio of the scratch force increased gradually with the increase in the amplitude. When scratching the Si-face of SiC polytypes, the reduction ratio of the F_x_ of -SiC was the largest, and when the amplitude was greater than or equal to 3 nm, the reduction ratio of F_z_ of 3C-SiC was the smallest, and there was little difference between 4H- and 6H-SiC. With the increase in the amplitude, the reduction ratio of F_x_ of 3C-, 4H- and 6H-SiC increased by 39.01%, 37.80% and 39.00%, respectively; the reduction ratio of F_z_ of 3C-, 4H- and 6H-SiC increased by 32.16%, 38.43% and 34.60%, respectively. When scratching the C-face of SiC polytypes, the F_x_ reduction ratio of 3C-SiC was the largest, the F_z_ reduction ratio of 3C-SiC was the largest and the F_z_ reduction ratio of 4H-SiC was the smallest. With the increase in the amplitude, the reduction ratio of F_x_ of 3C-, 4H- and 6H-SiC increased by 37.28%, 38.94% and 36.81%, respectively; the reduction ratio of F_z_ of 3C-, 4H- and 6H-SiC increased by 31.05%, 34.66% and 29.46%, respectively.

**Figure 4:**
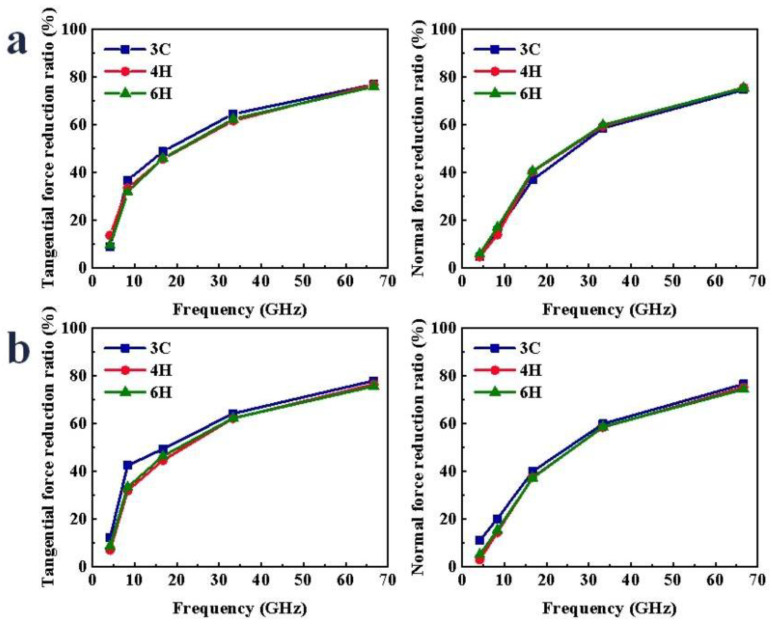
Variation in scratch force reduction ratio of SiC polytypes with different amplitudes: (**a**) Si-face and (**b**) C-face of SiC.

In the process of VS, the variations of scratch force under different frequencies are shown in Figure S3. With the increase in frequency and the more intensive the fluctuations in abrasive grain trajectory, the more frequent the repeated scratching of abrasive grain on workpiece atoms, so the obstruction in the direction of abrasive grain movement was reduced, and the scratch force required to remove workpiece atoms was small. This meant that the F_x_ and F_z_ were significantly reduced. As shown in Figure 4, during the VS process, as the frequency increased, the force reduction ratio increased. It was found that the difference in the force reduction ratio of the three crystal structures was very small. There was also a slight difference in the magnitude of the force between the C- and Si-faces, but the overall trends were the same. When scratching the Si-face of SiC polytypes, with the increase in the frequency, the reduction ratio of F_x_ of 3C-, 4H- and 6H-SiC increased by 67.90%, 63.19% and 66.51%, respectively; the reduction ratio of F_z_ of 3C-, 4H- and 6H-SiC increased by 70.00%, 70.85% and 69.61%, respectively. When scratching the C-face of SiC polytypes, with the increase in the frequency, the reduction ratio of F_x_ of 3C-, 4H- and 6H-SiC increased by 65.87%, 69.41% and 66.99%, respectively; the reduction ratio of F_z_ of 3C-, 4H- and 6H-SiC increased by 65.49%, 72.16% and 69.37%, respectively.

**Figure 5:**
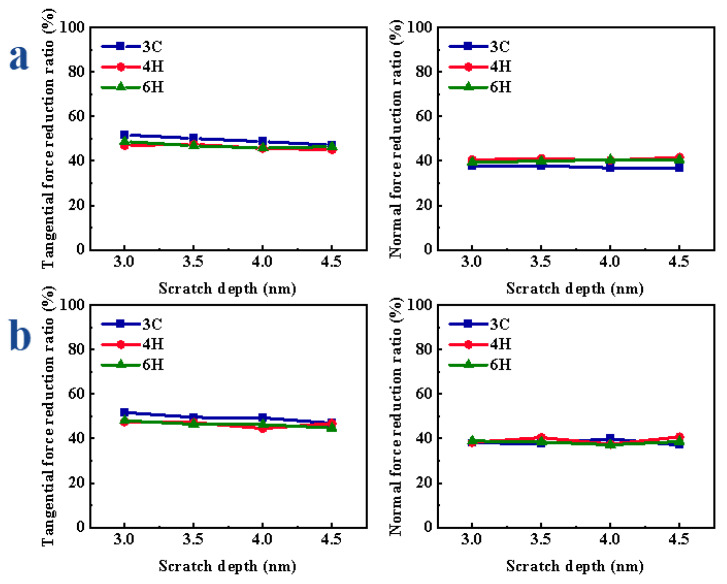
Variation in scratch force reduction ratio of SiC polytypes with different depths: (**a**) Si-face and (**b**) C-face of SiC.

The variations of scratch force under different depths are shown in Figure S4. In the process of VS, with the increase in scratch depth, the greater the obstacles in the process of material removal, so the F_x_ and F_z_ gradually increased. Compared with CS, the F_x_ and F_z_ of VS were significantly reduced and the F_y_ increased. When the scratch depth was different, the reduction ratio of the scratch force did not show an obvious change with the increase in the scratch depth. From Figure 5a, when scratching the Si-face of SiC polytypes, the reduction ratio of the F_x_ of 3C-SiC was the largest, while the reduction ratio of the F_z_ of 3C-SiC was the smallest. With the increase in the scratch depth, the reduction ratio of F_x_ of 3C-, 4H- and 6H-SiC changed by 4.60%, 2.38% and 3.30%, respectively; the reduction ratio of F_z_ of 3C-, 4H- and 6H-SiC changed by 0.99%, 1.31% and 1.09%, respectively. As shown in Figure 5b, when scratching the C-face of SiC polytypes, the F_x_ reduction ratio of 3C-SiC was the largest, and there was little difference between the F_z_ reduction ratio of 3C-, 4H- and 6H-SiC. With the increase in the scratch depth, the reduction ratio of F_x_ of 3C-, 4H- and 6H-SiC changed by 4.81%, 2.91% and 3.30%, respectively; the reduction ratio of F_z_ of 3C-, 4H- and 6H-SiC changed by 2.90%, 3.30% and 1.75%, respectively.

**Figure 6:**
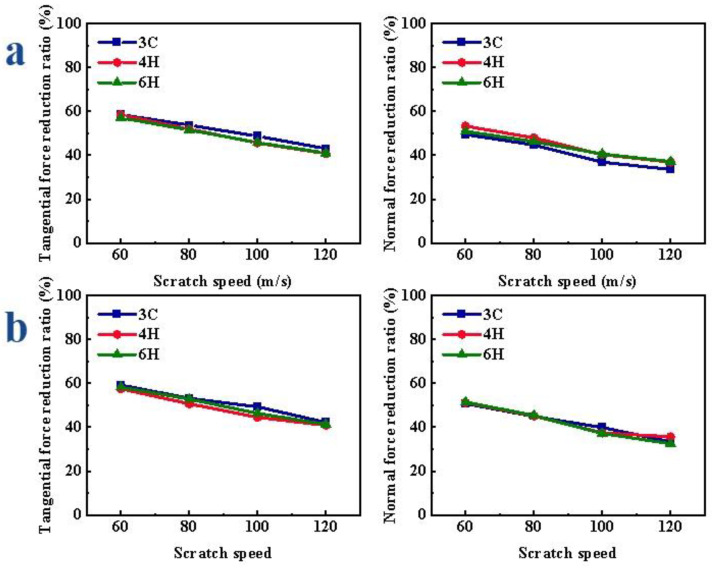
Variation in scratch force reduction ratio of SiC polytypes with different speeds: (**a**) Si-face and (**b**) C-face of SiC.

The variations of scratch force under different speeds are shown in Figure S5. In the CS, the F_x_ and F_z_ decreased with the increase in scratch speed; in the VS, with the increase in scratch speed, the number of vibrations of abrasive grain in the same scratch distance decreased, so the F_x_ and F_z_ increased gradually; compared with CS, the F_x_ and F_z_ of VS were significantly reduced, and the F_y_ was increased. When the speed was different, the reduction ratio of the scratch force gradually decreased with the increase in the speed. As shown in Figure 6a, when scratching the Si-face of SiC, the reduction ratio of the F_x_ of 3C-SiC was the largest; the reduction ratio of F_z_ of 3C-SiC was relatively small. With the increase in the scratch speed, the reduction ratio of F_x_ of 3C-, 4H- and 6H-SiC changed by 15.48%, 16.66% and 16.91%, respectively; the reduction ratio of F_z_ of 3C-, 4H- and 6H-SiC increased by 4.59%, 15.49% and 18.97%, respectively. When scratching the C-face of SiC polytypes, as shown in Figure 6b, the F_x_ reduction ratio of 3C-SiC was relatively the largest, and there was no obvious difference in the reduction ratio of F_z_ of different crystal structures. With the increase in the scratch speed, the reduction ratio of F_x_ of 3C-, 4H- and 6H-SiC decreased by 16.99%, 16.06% and 16.91%, respectively; the reduction ratio of F_z_ of 3C-, 4H- and 6H-SiC decreased by 17.43%, 13.77% and 18.97%, respectively.

### 3.2. Comparison of Amorphous Layer

**Figure 7:**
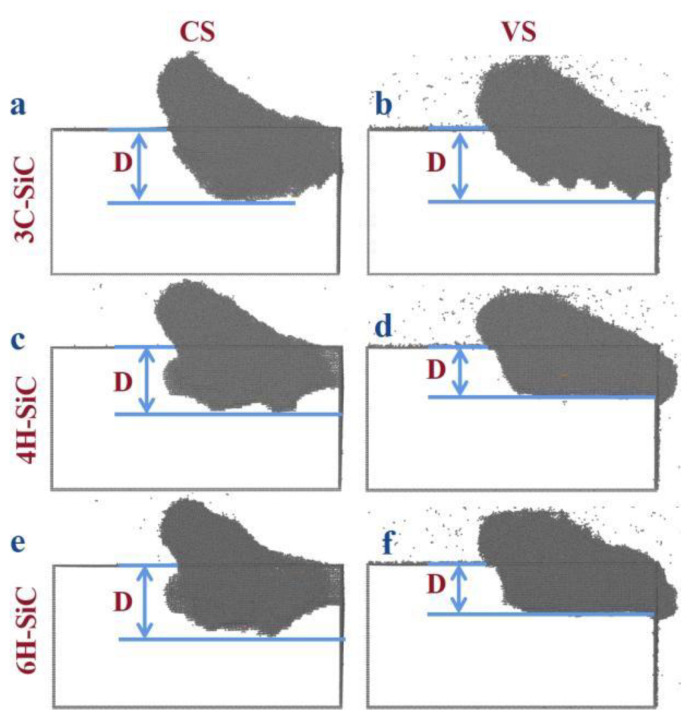
The amorphous atomic layer at the scratch distance of 15 nm of (**a**,**b**) 3C-SiC, (**c**,**d**) 4H-SiC and (**e**,**f**) 6H-SiC under CS and VS.

**Figure 8:**
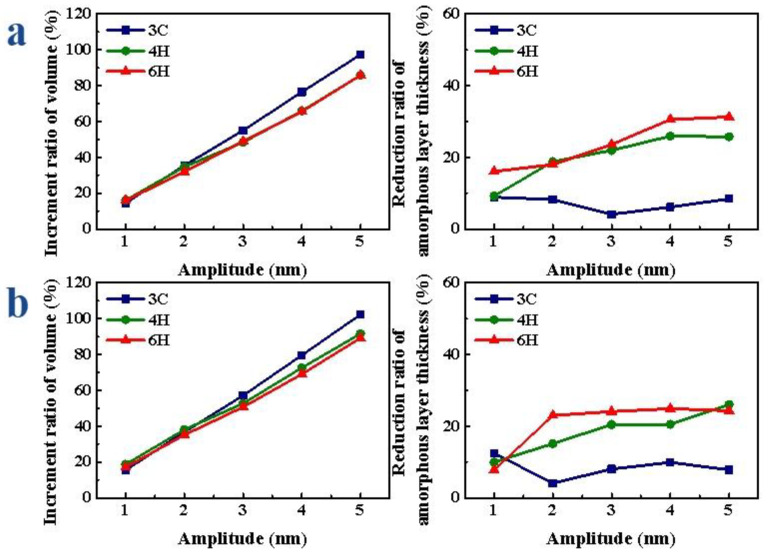
Amorphous atoms’ volume increment and amorphous atoms’ layer thickness reduction ratio of SiC polytypes with different amplitudes: (**a**) Si-face and (**b**) C-face of SiC.

In the process of machining, the original perfect lattice was destroyed, resulting in the amorphous layer due to the extrusion of abrasive grain on the workpiece material. The D is closely related to the damaged layer after machining. When the amorphous layer forms a certain depth, this will lead to subsurface damage. The decrease in amorphous layer thickness can explain the decrease in the damage layer, to a certain extent. The V represents the volume of the machining influenced area, which reflects the processing efficiency, to a certain extent. Through the thickness and removal volume of amorphous layer, the machining effect can be evaluated from two aspects: damage thickness and removal efficiency. In the MD simulation, the identify diamond structure and dislocation extraction algorithm (DXA) modules in OVITO were used to analyze the crystal structure. The part that is inconsistent with the original complete periodic lattice structure of the crystal is the amorphous layer, as shown in the gray area in Figure 7. The D is defined as the distance from the atom of the amorphous layer directly below the abrasive grain to the workpiece surface. The volume of the amorphous layer is expressed by the number of atoms in the amorphous layer. In Figure 8, it can be seen that the morphologies of SiC polytypes’ amorphous layers were different. The morphologies of the amorphous layers of 4H- and 6H-SiC were relatively close. In the VS process, the thickness significantly decreased and the bottom of the amorphous layers was flat. The amorphous layer of 3C-SiC had a special shape, especially after VS, where the bottom presented a sawtooth shape.

When the amplitude was different, with the increase in the amplitude, the V gradually increased, the D gradually decreased, the decreasing amplitude of 4H- and 6H-SiC gradually increased and the decreasing amplitude of 3C-SiC did not change much. As shown in Figure 8a, when scratching the Si-face of SiC polytypes, the volume increment ratio of the amorphous layer of 3C-SiC was the largest, and the thickness reduction ratio was the smallest. With the increase in the amplitude, the increment ratio of V of 3C-, 4H- and 6H-SiC increased by 82.73%, 69.67% and 69.77%, respectively; the reduction ratio of D of 3C-, 4H- and 6H-SiC increased by 4.77%, 16.70% and 15.17%, respectively. When scratching the C-face of SiC polytypes, as shown in Figure 8b, the increment ratio of V of 3C-SiC was the largest, followed by 4H-SiC, and 6H-SiC was the smallest; the reduction ratio of D of 6H-SiC was slightly greater than that of 4H-SiC and the minimum of 3C-SiC. With the increase in the amplitude, the increment ratio of V of 3C-, 4H- and 6H-SiC increased by 86.73%, 72.87% and 72.08%, respectively; the reduction ratio of D of 3C-, 4H- and 6H-SiC increased by 8.31%, 16.04% and 17.13%, respectively. Therefore, whether C-face or Si-face, the volume increment ratio of 3C-SiC was the largest, but the reduction ratio of D was the smallest.

**Figure 9:**
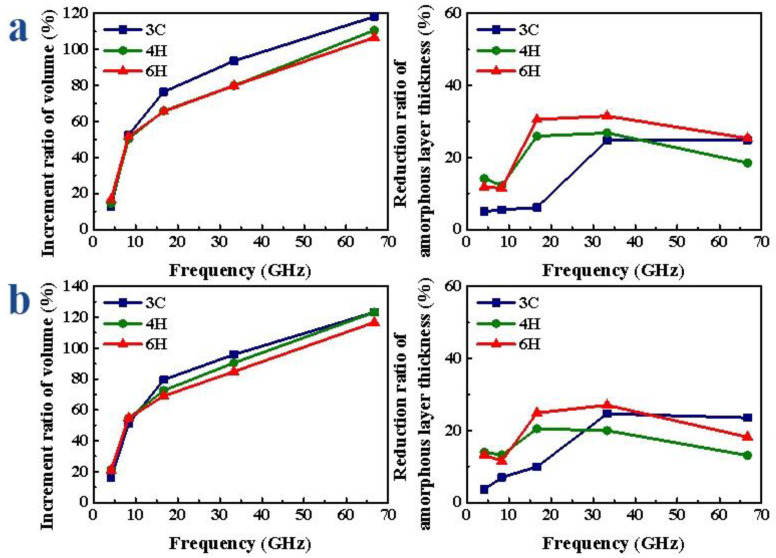
Amorphous atoms’ volume increment and amorphous atoms’ layer thickness reduction ratio of SiC polytypes with different frequencies: (**a**) Si-face and (**b**) C-face of SiC.

When the frequency was different, with the increase in frequency, the V gradually increased, the increment ratio of V gradually increased, the D gradually decreased and the decrease range increased first and then tended to be flat. As shown in Figure 9a, when scratching the Si-face of SiC polytypes, the volume increment ratio of the amorphous layer of 3C-SiC was the largest, and the thickness reduction ratio was relatively small. With the increase in frequency, the increment ratio of V of 3C-, 4H- and 6H-SiC increased by 105.25%, 96.11% and 106.63%, respectively; the reduction ratio of D of 3C-, 4H- and 6H-SiC increased by 19.78%, 14.60% and 25.33%, respectively. When scratching the C-face of SiC polytypes, as shown in Figure 9b, the volume increment ratio of the amorphous layer of 3C-SiC was the largest, followed by 4H-SiC, and 6H-SiC was the smallest; when the frequency was small, the reduction ratio of D of 6H-SiC was slightly greater than that of 4H-SiC, and 3C-SiC was the smallest. When the frequency increased to a certain extent, the reduction ratio of D of 3C-SiC was greater than that of 4H- and 6H-SiC. With the increase in frequency, the increment ratio of V of 3C-, 4H- and 6H-SiC increased by 107.22%, 101.98% and 116.69%, respectively; the reduction ratio of D of 3C-, 4H- and 6H-SiC increased by 21.06%, 7.41% and 18.18%, respectively.

**Figure 10:**
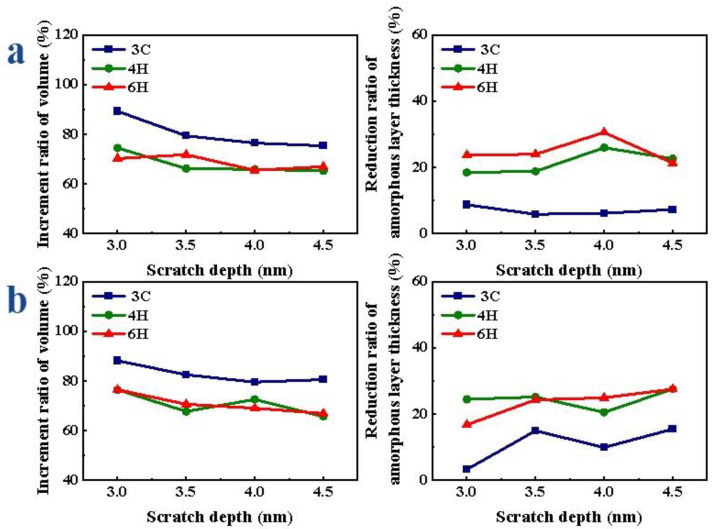
Amorphous atoms’ volume increment and amorphous atoms’ layer thickness reduction ratio of SiC polytypes with different depths: (**a**) Si-face and (**b**) C-face of SiC.

After applying vibration, the volume of the amorphous layer significantly increased and the thickness decreased at the same scratch depth. With the increase in the depth, the increment ratio of the V gradually decreased, and the reduction ratio of D did not change much. As shown in Figure 10a, when scratching the Si-face of SiC polytypes, the volume increment ratio of the amorphous layer of 3C-SiC was the largest, and the thickness reduction ratio was the smallest; the difference between the volume increment ratio of the amorphous layer of 4H- and 6H-SiC was not obvious, while the reduction ratio of the amorphous layer thickness of 4H-SiC was slightly smaller than that of 6H-SiC. With the increase in the scratch depth, the increment ratio of V of 3C-, 4H- and 6H-SiC changed by 13.90%, 9.19% and 6.29%, respectively; the reduction ratio of D of 3C-, 4H- and 6H-SiC increased by 2.87%, 7.45% and 9.25%, respectively. When scratching the C-face of SiC polytypes, as shown in Figure 10b, the change trends were basically the same as those of the Si-face. With the increase in the scratch depth, the increment ratio of V of 3C-, 4H- and 6H-SiC changed by 8.60%, 10.76% and 9.57%, respectively; the reduction ratio of D of 3C-, 4H- and 6H-SiC increased by 12.16%, 7.07% and 10.77%, respectively.

**Figure 11:**
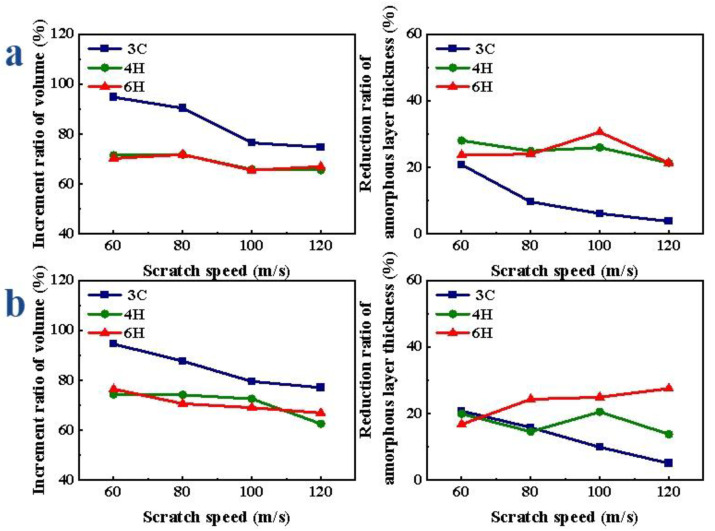
Amorphous atoms’ volume increment and amorphous atoms’ layer thickness reduction ratio of SiC polytypes with different speeds: (**a**) Si-face and (**b**) C-face of SiC.

After applying vibration, the V obviously increased and the thickness decreased at the same speed. With the increase in scratch speed, the increment ratio of V gradually decreased, the decrease range of D of 4H-SiC and 6H-SiC changed little and the decrease range of 3C-SiC gradually decreased. As shown in Figure 11a, when the Si face of SiC polytypes was scratched, the volume increment ratio of the amorphous layer of 3C-SiC was the largest and the thickness reduction ratio was the smallest; there was little difference in the volume increment ratio between 4H- and 6H-SiC, and there was little difference in the thickness reduction ratio of the amorphous layer between 4H- and 6H-SiC. With the increase in scratch speed, the increment ratio of V of 3C-, 4H- and 6H-SiC changed by 20.03%, 6.14% and 12.96%, respectively; the reduction ratio of D of 3C-, 4H- and 6H-SiC increased by 17.00%, 6.69% and 16.72%, respectively. When the C-face of SiC polytypes was scratched, as shown in Figure 11b, the change trends were basically consistent with that of Si-face. With the increase in scratch speed, the increment ratio of V of 3C-, 4H- and 6H-SiC changed by 17.42%, 11.74% and 10.13%, respectively; the reduction ratio of D of 3C-, 4H- and 6H-SiC increased by 15.69%, 6.71% and 7.33%, respectively.

## 4. Conclusions

By using the molecular dynamics method (MD) to simulate the scratching process of different crystal faces of SiC polytypes, the differences between convention scratch (CS) and vibration-assisted scratch (VS) were compared and analyzed from the perspectives of scratch force and amorphous layer, as well as the differences in the vibration application effects on different crystal structures. The following conclusions are drawn:

(1) Vibration-assisted scratch of SiC polytypes has the advantages of reducing the scratch force, increasing the volume and reducing the thickness of the amorphous layer; this means that the material removal rate is improved, and the damage degree is reduced.
(2) Increasing the amplitude and frequency improves the machining effect. The changes in scratch depth and speed do not notably improve the effect of VS.
(3) The effects of vibration on SiC with different crystal structures are different. The effects of 4H- and 6H-SiC in the hexagonal structure are close, and the effect of 3C-SiC makes an obvious distinction between that of 4H- and 6H-SiC. The morphology of the 3C-SiC amorphous layer structure after vibration is obviously different from 4H- and 6H-SiC, showing a sawtooth shape.
(4) The effects of vibration on the SiC of different crystal faces are different, but the difference is not significant. Under different vibration parameters and machining parameters, the variation trends of the reduction ratio of the scratch force and the increment ratio of volume and the reduction ratio of the thickness of the amorphous-layer in the VS of SiC on different crystal planes are consistent.
(5) The MD simulation in this paper provides a comparison of the effects of vibration applied to SiC polytypes under different machining and vibration parameters, which provides a basis for the high-efficiency and precision machining of SiC using vibration-assisted grinding technology.

## Supplementary Materials

The following are available online at https://www.mdpi.com/article/10.3390/mi13040640/s1, Figure S1: Schematic diagram of atomic arrangement of SiC polytypes (3C-, 4H- and 6H-SiC) [18], "h" and "k" represent the sites of hexagonal and cubic. Figure S2: Variation of scratch force of SiC polytypes with different amplitudes: (a) Si-face and (b) C-face of SiC. Figure S3: Variation of scratch force of SiC polytypes with different frequencies: (a) Si-face and (b) C-face of SiC. Figure S4: Variation of scratch force of SiC polytypes with different depths: (a) Si-face and (b) C-face of SiC. Figure S5: Variation of scratch force of SiC polytypes with different speeds: (a) Si-face and (b) C-face of SiC.
