# Supplementary material for: Comparison of Vibration-Assisted Scratch Characteristics of SiC Polytypes (3C-, 4H- and 6H-SiC)
Source: Micromachines (Basel). 2022 Apr 18;13(4):640. doi: 10.3390/mi13040640 (PMC9030126; doi:10.3390/mi13040640)
Supplement: Supplementary file 1 [file micromachines-13-00640-s001.zip › micromachines-1667367-supplementary.pdf]

Supplementary Materials: Comparison of Vibration-Assisted Scratch Characteristics of SiC Polytypes (3C-, 4H- and 6H-SiC)

Wuqing Lin [1,2,3], Zhongwei Hu [1,2,*], Yue Chen [1,2], Yuqiang Zhang [1,2], Yiqing Yu [2], Xipeng Xu [1,2] and Jie Zhang [3]

[1] Institute of Manufacturing Engineering, Huaqiao University, Xiamen 361021, China; vince_lin@sanan-ic.com (W.L.); chenyue@stu.hqu.edu.cn (Y.C.); 21011080019@stu.hqu.edu.cn (Y.Z.); xpxu@hqu.edu.cn (X.X.)
[2] Institute of Mechanical Engineering and Automation, Huaqiao University, Xiamen 361021, China; yyqing@hqu.edu.cn
[3] Hunan Sanan Semiconductor Co., Ltd., Changsha 410000, China; jack_zhang@sanan-ic.com
* Correspondence: huzhongwei@hqu.edu.cn

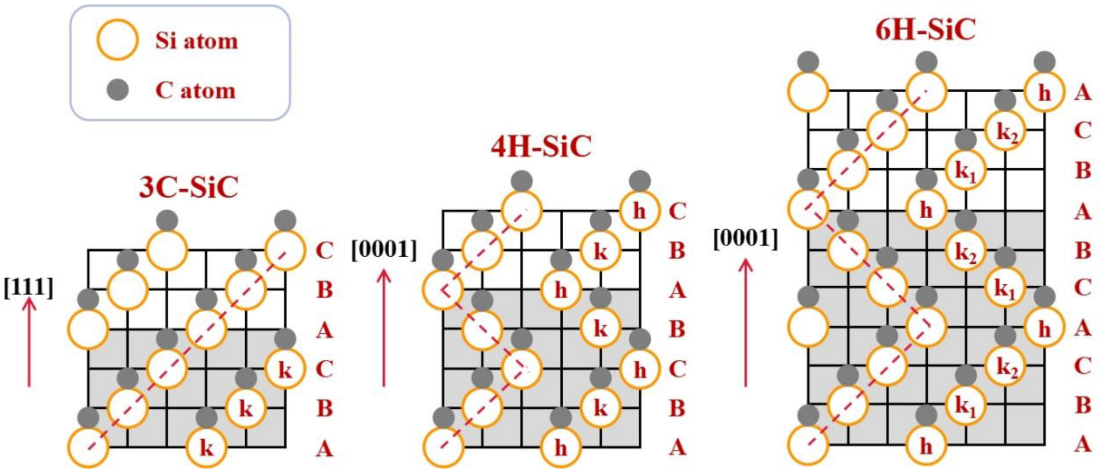

Figure S1. Schematic diagram of atomic arrangement of SiC polytypes (3C-, 4H- and 6H-SiC) [15], "h" and "k" represent the sites of hexagonal and cubic.

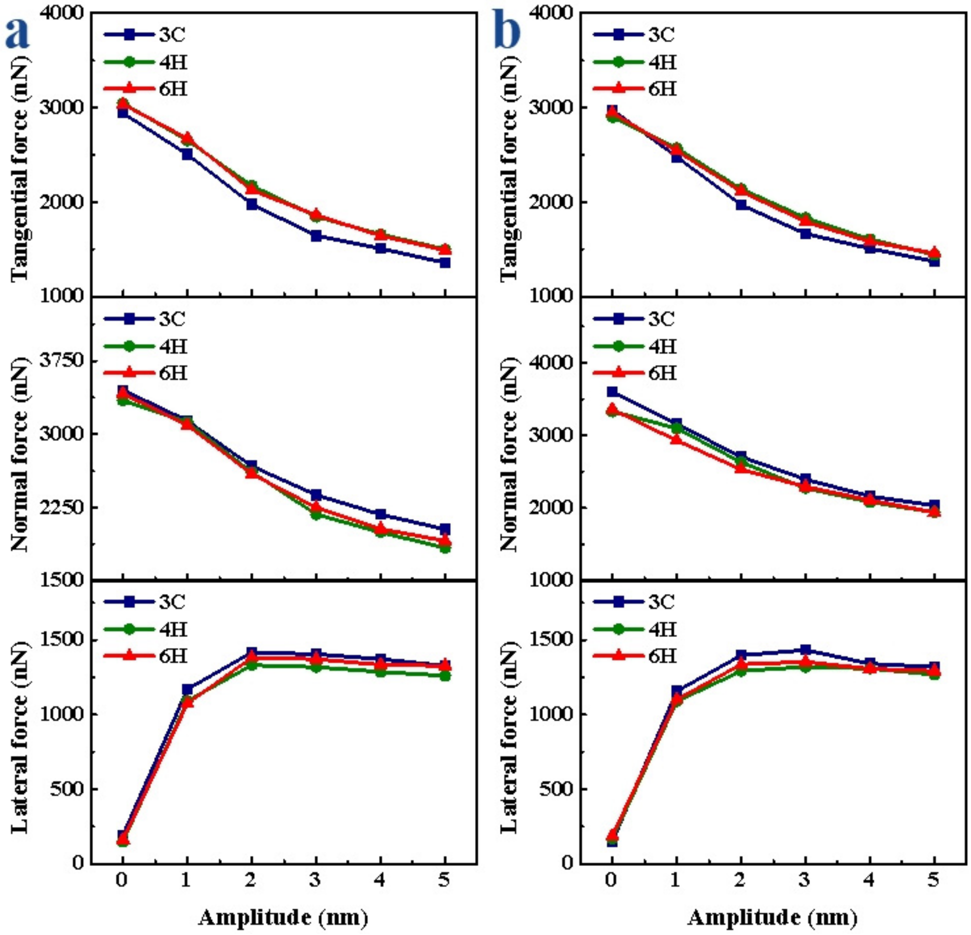

Figure S2. Variation of scratch force of SiC polytypes with different amplitudes: (**a**) Si-face and (**b**) C-face of SiC.

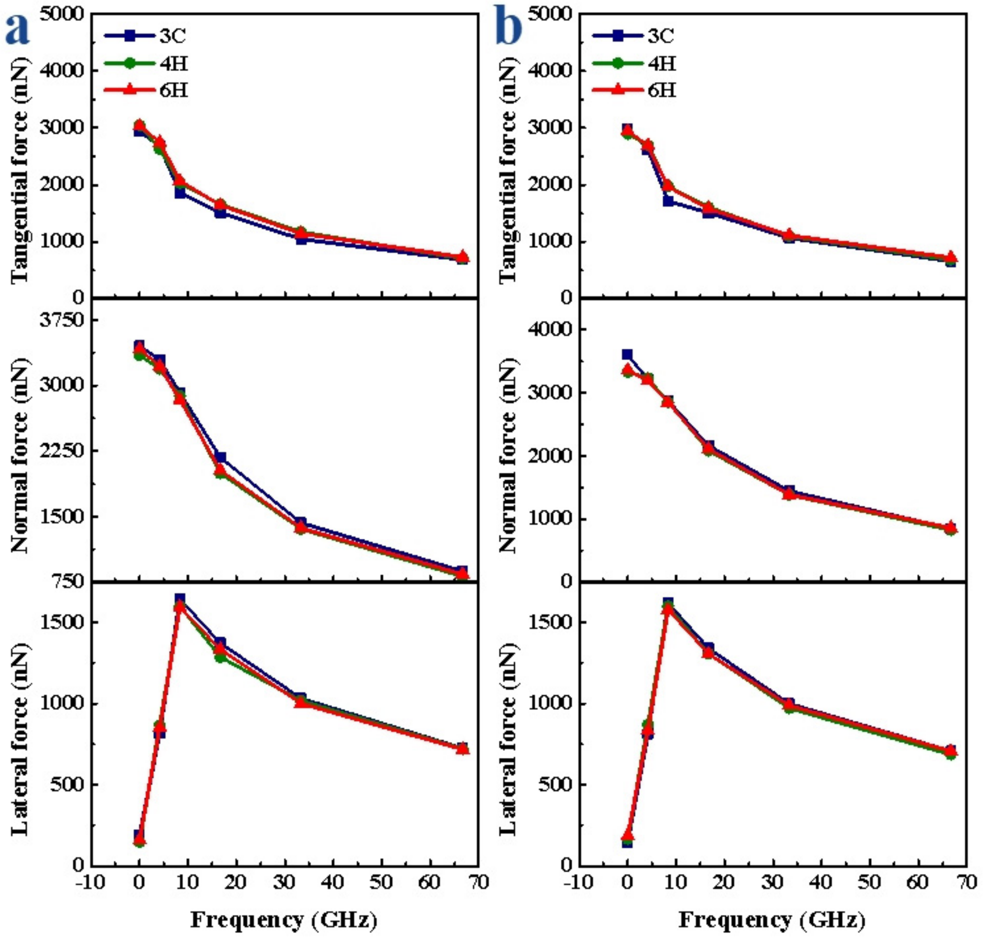

Figure S3. Variation of scratch force of SiC polytypes with different frequencies: (**a**) Si-face and (**b**) C-face of SiC.

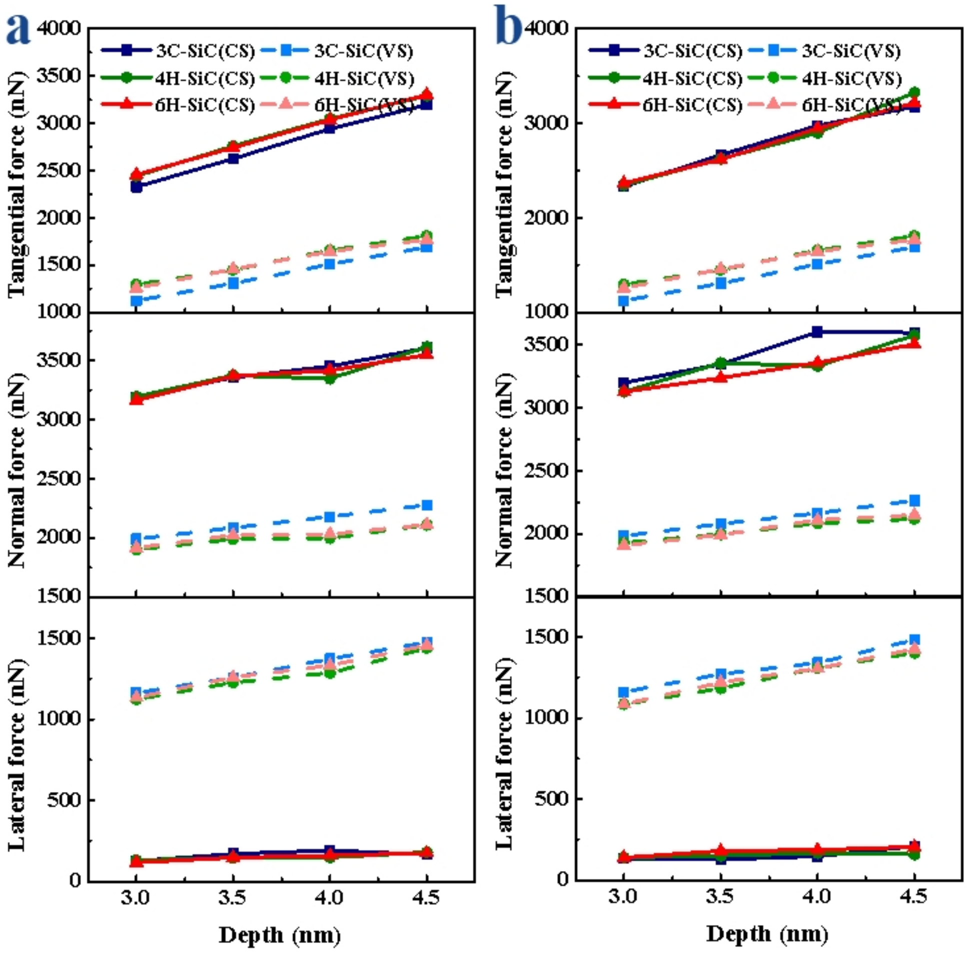

Figure S4. Variation of scratch force of SiC polytypes with different depths: (**a**) Si-face and (**b**) C-face of SiC.

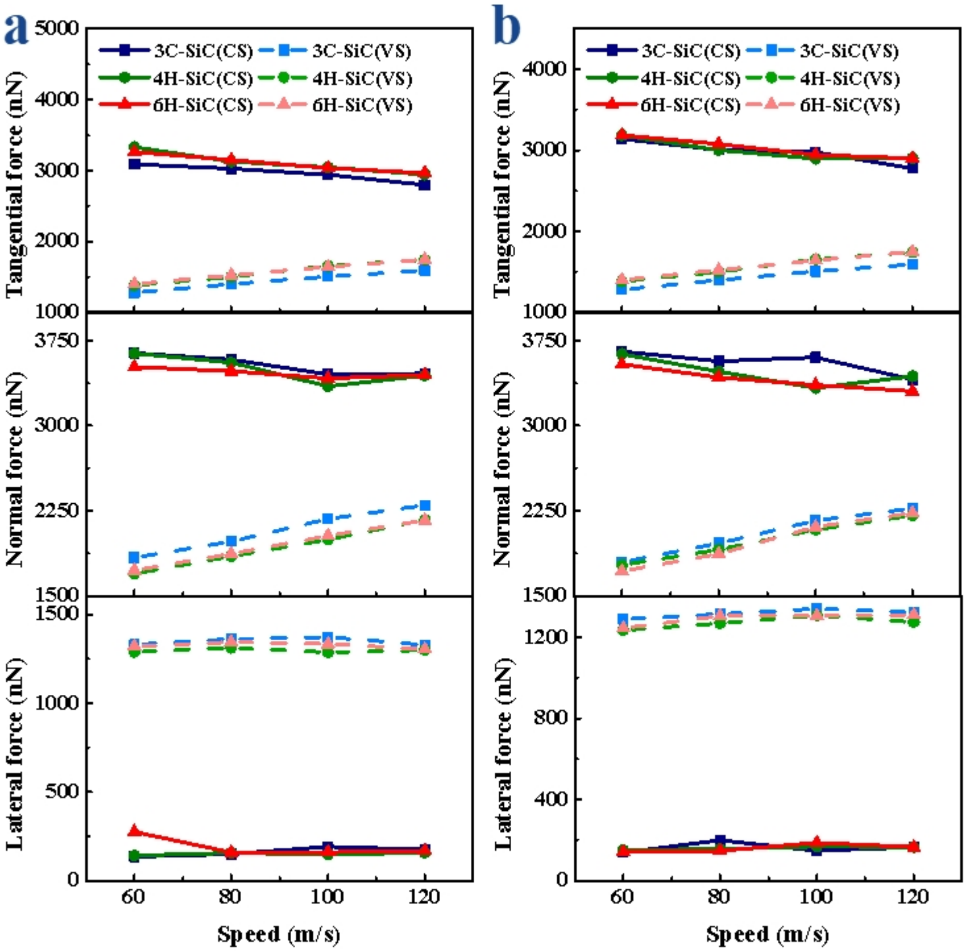

Figure S5. Variation of scratch force of SiC polytypes with different speeds: (**a**) Si-face and (**b**) C-face of SiC.
